# Intravenous Tranexamic Acid Given at Femoral Fragility Fracture Surgery Reduces Blood Transfusion Requirements Fourfold

**DOI:** 10.1007/s00268-022-06886-2

**Published:** 2023-02-01

**Authors:** Matilda F. R. Powell-Bowns, Rhys K. Olley, Conor McCann, James R. Balfour, Caitlin M. Brennan, Jasmine Peh, Andrew D. Duckworth, Chloe E. H. Scott

**Affiliations:** 1 Specialist Trainee in Trauma and Orthopaedics, Royal Infirmary of Edinburgh, Little France Cres, Edinburgh, Edinburgh, EH164SA Scotland UK; 2 University of Edinburgh, Edinburgh, Scotland UK

## Abstract

**Aims:**

This study aims to determine whether intraoperative intravenous (IV) tranexamic acid (TXA) affects blood loss following the surgical management of femoral fragility fractures (FFF).

**Methods:**

This was a single centre (university teaching hospital) non-randomised case–control study. There were 361 consecutive patients with FFF admitted over a 4-month period were included (mean age 81.4yrs; mean BMI 23.5; 73.7% female). Patient demographics, comorbidities, preoperative anticoagulation use, surgical management, intravenous TXA use, perioperative haemoglobin (Hb) and haematocrit, and requirement for blood transfusion were recorded. The primary outcome was postoperative blood transfusion requirement. Secondary outcomes included postoperative day one calculated blood loss (CBL) (using the Nadler and Gross formulae) and fall in Hb (percentage) from preoperative levels; and the incidence of thrombotic events and mortality up to 30 days.

**Results:**

Groups were well matched in terms of patient demographics, comorbidities, preoperative anticoagulation use, injury types and surgical management. Intravenous TXA 1 g given at the beginning of surgery at the discretion of the operating team: 178 (49%) received TXA and 183 (51%) did not. The requirement for postoperative blood transfusion was significantly less in the TXA group: 15/178 (8.4%) compared to 58/183 (31.7%) (*p* < 0.001; Chi square). TXA significantly reduced both the percentage fall in Hb (mean difference 4.3%, *p* < 0.001) and the CBL (mean difference −222 ml, *p* < 0.001). There was no difference in VTE (2 vs 1, *p* = 0.620) or other thrombotic events (2 vs 0, *p* = 0.244) between groups.

**Conclusion:**

1 g of intraoperative intravenous TXA during the surgical management of FFF was associated with reduced rate of transfusion, CBL and the percentage drop in HB. The use of TXA in this study was not randomised, so there could be un-quantifiable bias in the patient selection.

## Introduction

TXA is an inexpensive synthetic lysine analogue that acts as a competitive antagonist to plasminogen [2]. This reduces the concentration of active plasmin and increases the half-life of fibrin, making it an antifibrinolytic and clot stabilising agent [2]. TXA is used across many subspecialties to prevent bleeding [2–4]. Cochrane supports TXA use during urgent surgery [5].

Perioperative TXA use has repeatedly been found to reduce blood loss and the need for blood transfusion following elective arthroplasty [6–13]. A single dose of TXA has been shown to reduce the requirement for postoperative blood transfusion following elective orthopaedic procedures by as much as 42% [6–15]. This reduced need for transfusion improves patient outcomes [9, 15] and aids health care systems through reduced length of inpatient stay and transfusion costs [14–16].

Current United Kingdom national guidance recommends TXA use during primary arthroplasty procedures [16]. TXA use in fracture management remains unclear. The European Society of Anaesthetists does not recommend TXA use for patients requiring hip fracture surgery. Several randomised control trials have investigated the role of TXA use in hip fracture surgery [6, 11, 12, 17]. Of these RCTs, all have limitations diluting their clinical message. Despite this, the RCTs support the use of TXA in hip fracture surgery, suggesting reduces blood loss without increasing the rate of venous thromboembolism (VTE) [6, 11, 12, 17].

Hip fracture patients and those with other FFFs (distal femur; periprosthetic; and femoral shaft), share similar demographics, invariably require surgery and have comparable morbidity and mortality [18–20]. In the United Kingdom, there is a shift to consider all FFFs together under the same guidelines instead of concentrating on hip fractures alone [18–20]. Currently, TXA is not included in the UK national or US guidelines for hip fracture or FFF surgery [18, 21].

This study aims to determine whether intraoperative intravenous TXA affects blood loss following the surgical management of all FFF. The primary outcome measure was postoperative blood transfusion requirement.

Secondary outcomes included calculated blood loss (CBL), percentage drop in haemoglobin (Hb), early postoperative complications, 30-day mortality and an estimate of cost-effectiveness.

## Materials and methods

**Fig. 1:**
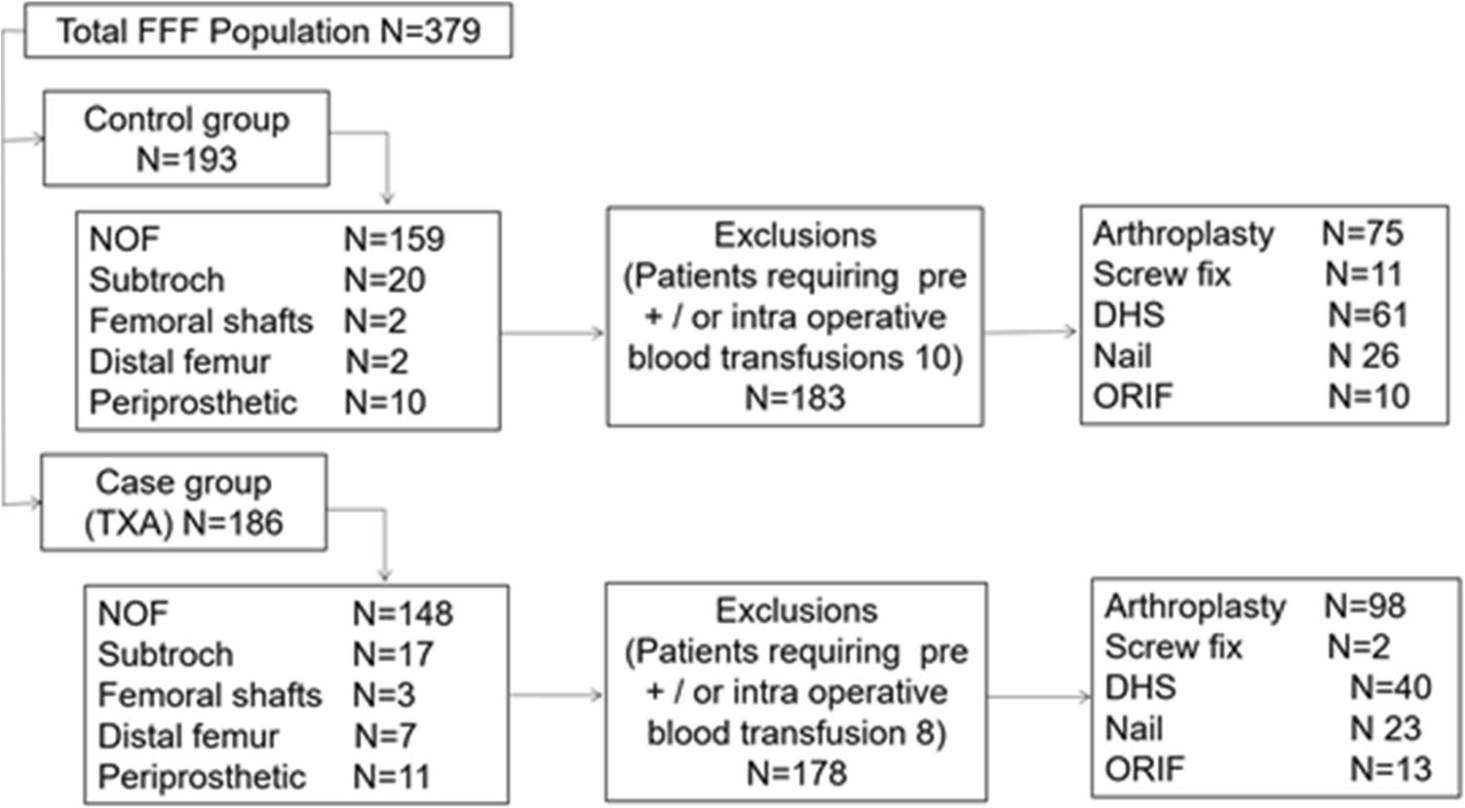
Details the participant journey within the study including: fracture type, TXA use, exclusions, and surgical management

From 1st of January 2020 to 30th April 2020, consecutive patients with FFFs attending our institution were identified prospectively on admission. Patients who were managed non-operatively, or who received preoperative or intraoperative blood transfusion were excluded (*n* = 18). This gave a study population of 361 patients with surgically managed FFFs including: intracapsular (IC) and extracapsular (EC) hip fractures, subtrochanteric fractures, femoral shaft fractures, distal femur fractures, and periprosthetic fractures involving the femoral component of hip or knee prostheses. Details of the patient cohort are presented in Fig. 1. Institutional approval was obtained from the NHS Lothian Quality improvement committee.

### TXA administration

Administration of intravenous TXA was given at the discretion of the operating team. Surgery was performed/supervised by 21 consultant orthopaedic surgeons: 116/361 (32%) patients were treated by surgeons who regularly perform elective arthroplasty with routine administration of intravenous TXA; 59/361 (16%) by trauma surgeons who routinely give TXA; and 186/361 (51.5%) by trauma surgeons who do not routinely administer TXA. Patients receiving TXA were given 1 g intravenous intraoperatively, with 178 patients receiving TXA (49% cases) and 183 patients who did not (51%). Any patient taking oral anticoagulants on admission (direct-acting-oral-anticoagulant (DOAC), warfarin, or heparin) had these withheld. Patients taking apixaban underwent surgery from 24 h after the last apixaban dose (if patient had normal renal function). Patients, taking warfarin, had this reversed with vitamin K until the INR was ≤ 1.4. All patients received VTE prophylaxis with subcutaneous Dalteparin (2500units for patients ≤ 50 kg, 5000 units for patients > 50 kg) at > 6–8 h postoperatively and for 6 weeks postoperatively. Where patients had previously taken oral anticoagulants prior to surgery, these were reintroduced 48 h postoperatively and Dalteparin stopped.

### Outcomes

Operation notes, anaesthetic charts and prescription charts were examined throughout admission and the following data were recorded: demographic data, Charlson Comorbidity index [22], American Society of Anaesthesiologists (ASA) score [22], BMI, injury, operation type, preoperative and postoperative day 1 haemoglobin (Hb, g/L) and haematocrit (Hct), requirement for blood transfusion within 5 days of surgery, number of units transfused, and immediate postoperative complications. Electronic patient records were reviewed after discharge to record 30-day mortality and early (< 6 weeks) postoperative complications.

The indications for postoperative blood transfusion were as follows: patients with a significant cardiac history and an Hb  < 100 g/L, all other patients with an Hb < 80 g/L who were symptomatic of anaemia. The number of units was at the clinicians’ discretion. Hb drop was defined as the percentage fall between preoperative and postoperative day 1 values. Estimated blood volume (EBV) (mls) was calculated using the Nadler equation according to patient height and weight [23]. Calculated blood loss (CBL) (mls) was determined using the Gross [24] formula based on EBV and changes in haematocrit.

Nadler formulae:

EBV (Male) = 604 + 0.0003668[(height (cm)]^3 + 32(weight(kg)).

EBV (Female) = 183 + 0.000356[(height (cm)]^3 + 32(weight(kg)).

Gross formula:

CBL = EBV × [(Hct preop–Hct postop)/Hct Average].

### Costs

To estimate the cost-effectiveness of TXA administration, the following costs were taken from Cochrane data [24]: TXA IV 1 g (2 × 500 mg vials) £2.25 per patient; and a red blood cell transfusion £186 for the first and £165 for subsequent units [24]. The total cost of TXA (*n* = 178) for the study cohort was therefore £400.50. The median number of units transfused was 2, and thus, the total cost of a transfusion episode was assumed to be £351.

### Statistical analysis

Data were analysed using SPSS version 25.0. Categorical variables were analysed using Chi square or Fisher’s exact test. Continuous variables were analysed using the *T* test, with ANOVA used for multiple groups. Correlations were assessed for using Pearson’s test or Spearman’s Rho test. Nonparametric data were analysed using the Mann–Whitney U test. A *p* value of ≤ 0.05 was considered statistically significant. A post hoc power calculation using the defined rate of blood transfusion (8% in the case group (*n* = 178) and 32% in the control group (*n* = 163)) with an alpha of 0.05 a two-way analysis defined the power as 100%.

## Results

From January 2020 to May 2020, 379 consecutive FFF presented to the study centre. There were 18 patients that required a pre ± intraoperative blood transfusion. This gave a study population of 361 patients with 361 FFFs (Fig. 1). The mean age was 81.4 (32–101), the mean BMI was 23.5 (10–46) and 266 were female (73.7%). At presentation, 60 patients (16.6%) were anticoagulated with heparin, warfarin or a DOAC and 33 patients (9.1%) were on clopidogrel antiplatelet medication (Table 1).

**Table 1:** Summary of patient characteristics, fracture classification, surgical management and time to surgery for the two groups

Variable		Case TXA (*n* = 178)	Control No TXA (*n* = 183)	*p* value
Mean Age		81.5 (9.4)	81.3 (10.8)	0.815*
Mean BMI		23.6 (5.0)	23.5 (5.0)	0.807*
Female gender		128 [72]	139 [76]	0.395^
ASA	1	5 [3]	4 [2]	0.256^
	2	56 [31]	43 [23]	
	3	99 [56]	108 [59]	
	4	17 [10]	26 [14]	
Anticoagulation	Warfarin, heparin or DOAC	28 [16]	32 [17]	0.655^
	Clopidogrel	16 [9]	17 [9]	0.656^
Fracture type	Intracapsular	100 [56]	92 [50]	0.223^
	Extracapsular	40 [22]	59 [32]	
	Subtrochanteric	17 [9.5]	18 [10]	
	Femoral shaft	3 [1.7]	2 [1]	
	Periprosthetic	11 [6]	10 [5]	
	Distal	7 [4]	2 [1]	
Management	Hemiarthroplasty	86 [48]	70 [38]	0.012^
	Cannulated screws	2 [1]	11 [6]	
	DHS	40 [22]	61 [30]	
	THA	12 [7]	5 [3]	
	IMN	23 [13]	26 [14]	
	ORIF	13 [7]	10 [5]	
Median Days to surgery		1 (1–2)	1 (1–2)	0.740**

Mean (SD), number [%]. *Unpaired *T* test, ** Mann–Whitney *U* test, ^ Chi square

**Fig. 2:**
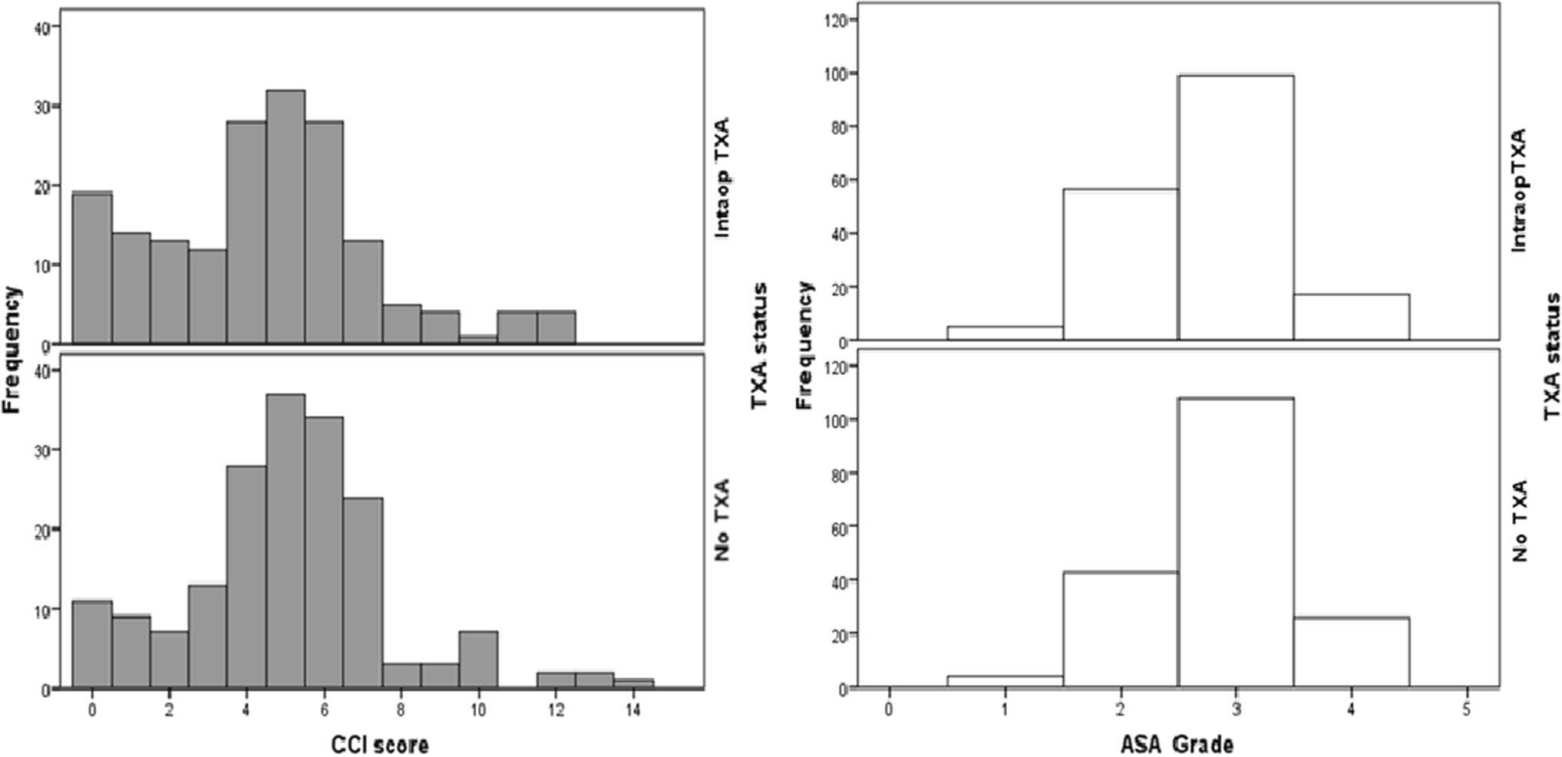
Displays histograms demonstrating the CCI and ASA scores of patients in each group according to TXA status

At the time of surgery, 178 patients were given 1 g intravenous TXA at the start of surgery (cases) and 183 were not given TXA (controls). Patient characteristics, anticoagulation, fracture types and operative management are detailed in Table 1. There were no significant differences in age, gender, BMI or pre-fracture anticoagulation between cases and controls preoperatively (Table 1). There was no significant difference in Charlson comorbidity index (CCI) or ASA grade between groups (*p* = 0.078, Chi square) (Fig. 2). Patients undergoing DHS were less likely to be given TXA and those undergoing THA were more likely to be given TXA (*p* = 0.012, Chi square). There was no significant difference in preoperative Hb, Hct and EBV between the TXA case and control groups (Table 2).

**Table 2:** Blood loss and transfusion requirements in TXA and no TXA groups

Variable	Case TXA (*n* = 178)	Control No TXA (*n* = 183)	Mean difference	*p* value
Preoperative Hb (g/L)	121.9 (16.1)	121.5 (15.0)	0.437	0.790*
95% CI	(119.63–124.1)	(119.0 to 123.5)		
Preoperative Hct (Ratio)	0.357 (0.044)	0.355 (0.044)	0.003	0.548*
95% CI	(0.35 to 0.36)	(0.35 to 0.36)		
EBV (ml)	4253 (666)	4161 (646)	91.3	0.187*
95% CI	(4154 to 4357)	(4064 to 4253)		
Postoperative Hct	0.304 (0.05)	0.287 (0.05)	0.018	0.001*
95% CI	(0.298 to 0.312)	(0.279 to 0.295)		
Postoperative Hb (g/L)	104 (16)	98.5 (17.8)	5.38	
95% CI	(101 to 106)	(95.8 to 101)		0.003*
Hb drop (%)	14.6 (8.9)	18.9 (10.3)	4.30	< 0.001**
95% CI	(7.9 to 9.8)	(17.4 to 20.6)		
CBL (ml)	692 (489)	914 (618)	221.5	< 0.001**
95% CI	(617 to 762)	(825 to 1014)		
Transfusion given	15 [8]	58 [32]		< 0.001^

Mean (SD), number [%]. * Unpaired *T* test, ** Mann–Whitney *U* test, ^ Chi square

*Hb* haemoglobin; *Hct* haematocrit; *EBV* estimated blood volume; *CBL* calculated blood loss

### Blood transfusion

The blood transfusion rate was significantly less in the TXA group at 8.4% (15/178) compared to the control group (58/183, 31.7%; *p* < 0.001). Where transfusion was performed, a median of 1 unit (IQR 1–2) was required in the TXA group compared to median 2 units (IQR1-2) in controls (*p* = 0.935, Mann–Whitney U test). A maximum of 3 units was transfused in the TXA group and 5 units in controls. TXA reduced the relative risk of blood transfusion by 73% compared to not giving TXA (RR 0.27, 0.16–0.45 95% CI, *p* < 0.001) (Table 2). When transfusion was indicated, there was no significant difference in the number of units transfused (*p* = 0.632) between TXA and control groups. The requirement for blood transfusion was significantly lower in the TXA case group for each type of surgery (Table 3).

**Table 3:** Blood loss and transfusion requirements by operation type

Variable	IC fracture surgery			DHS			Cephalomedullary		
	TXA (*n* = 101)	No TXA (*n* = 87)	*p* value	TXA (n = 40)	No TXA (*n* = 62)	*p* value	TXA (n = 23)	No TXA (*n* = 26)	*p* value
Hb drop (%)	11.3 (6.7)	14.4 (8.1)	0.005*	16.2 (7.3)	19.6 (9.9)	0.060*	24.9 (10.3)	30.1 (8.9)	0.064*
95%CI	10.0–12.7	12.8–16.3		13.8–18.5	17.1–22.1		20.7–29.2	26.8–33.4	
CBL (ml)	523 (375)	637 (429)	0.053*	729 (381)	945 (600)	0.030*	1220 (614)	1614 (628)	0.032*
95%CI	450–611	553–738		608–849	800–1090		963–1474	1395–1852	
Transfusion	5 [5]	12 [14]	0.035^	2 [5]	22 [35]	< 0.001^	7 [30]	16 [62]	0.029^
Number of red cell units (median, IQR)	2 (1–4)	2 (1–2)	0.393**	1	1 (1–3)	0.393**	2 (1–3)	2 (1–3)	1.0**

Mean (SD), number [%], median (IQR). * Unpaired *T* test, ** Mann–Whitney *U* test, ^ Chi square

*Hb* haemoglobin; *CBL* calculated blood loss; *IQR* interquartile range

### Calculated blood loss and percentage drop in haemoglobin

**Fig. 3:**
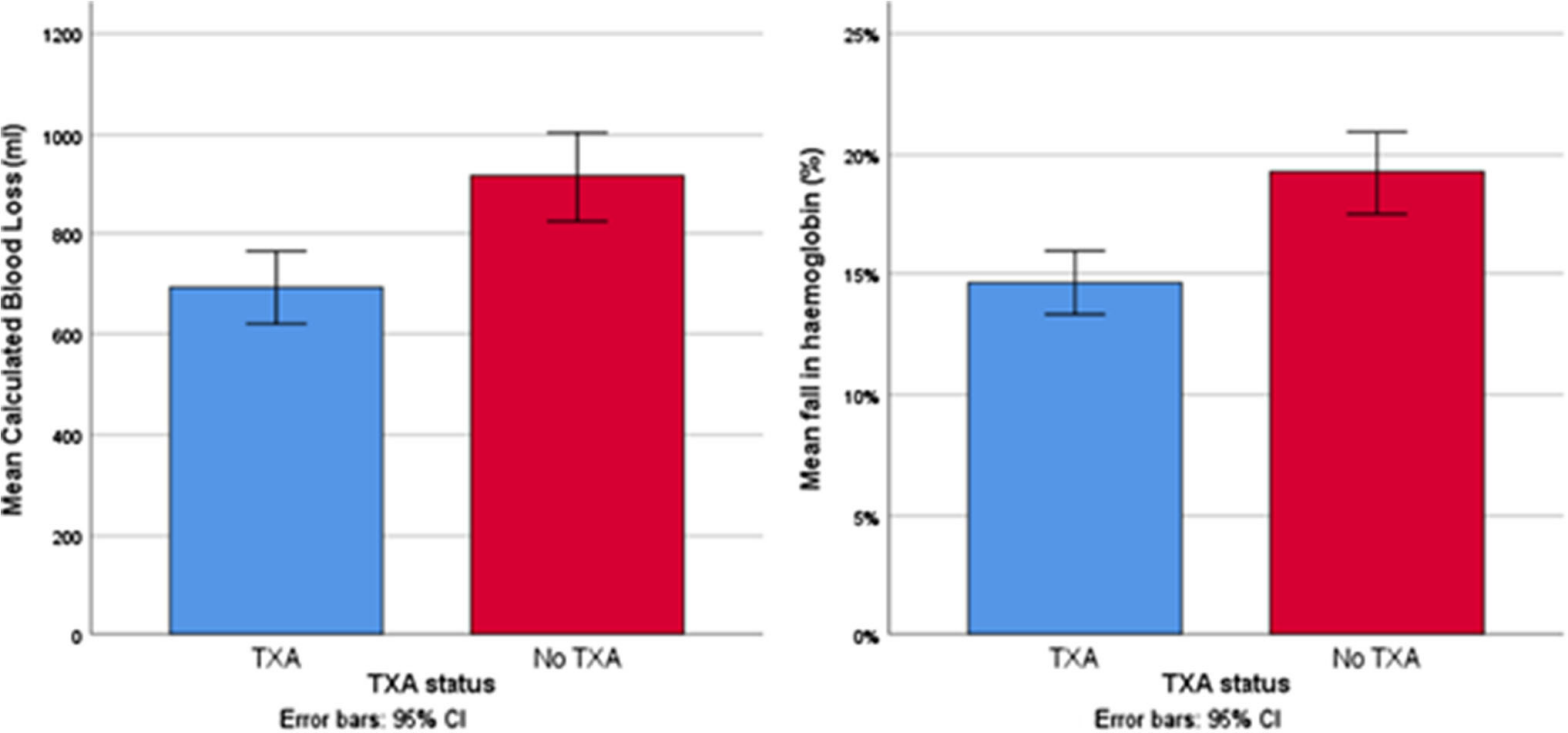
Calculated blood loss (CBL) and percentage fall in haemoglobin (Hb) following TXA at the time of femoral fragility fracture surgery and no TXA. Significant differences in both CBL (*p* < 0.001) and percentage fall in Hb (*p* < 0.001) were identified

**Fig. 4:**
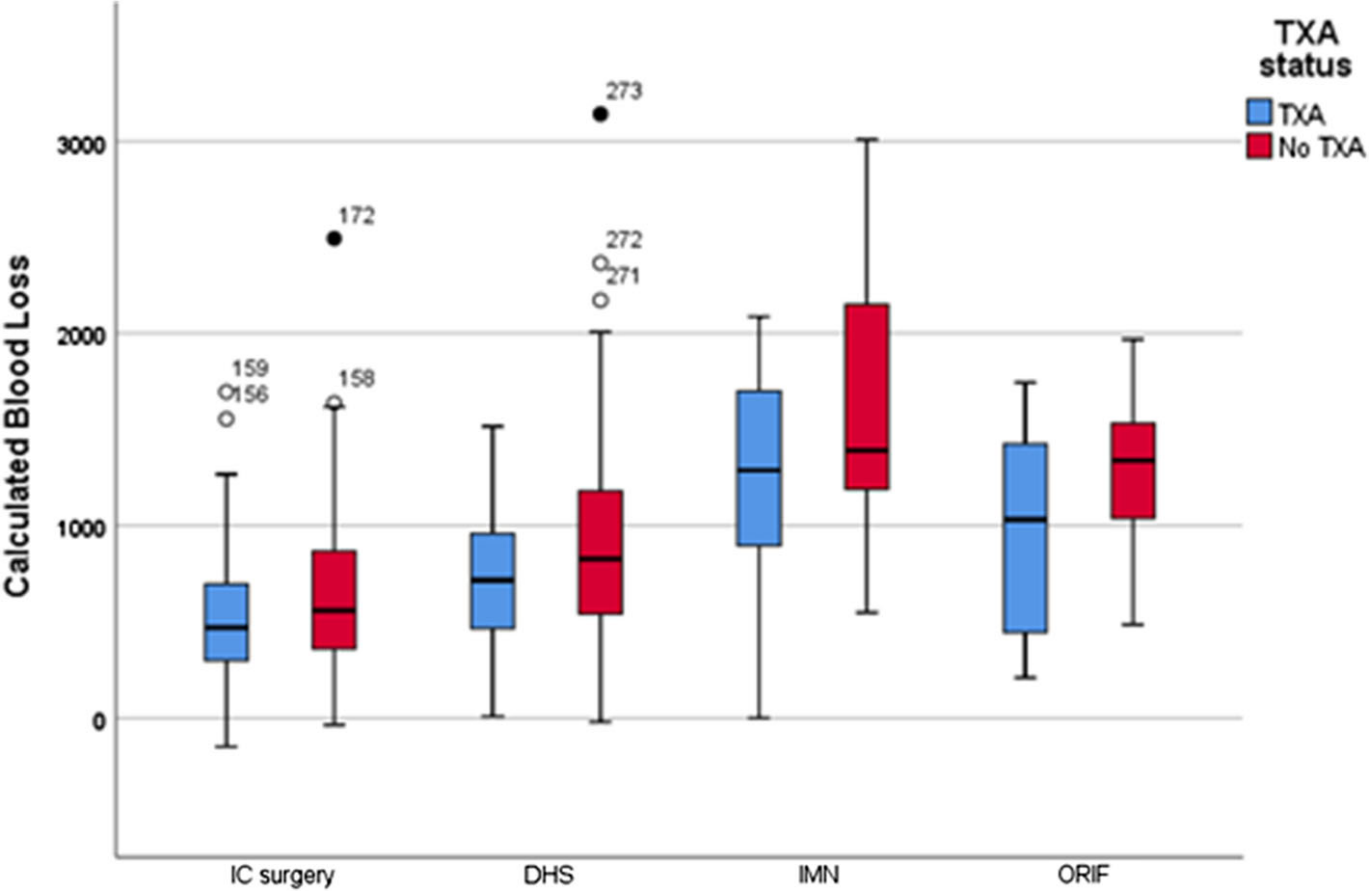
Calculated blood loss according to fracture surgery and TXA status. IC surgery = surgery for intracapsular hip fracture including cannulated screw fixation, hemiarthroplasty and total hip arthroplasty; DHS = dynamic hip screw; IMN = cephalomedullary nail; ORIF – open reduction and internal fixation of periprosthetic fracture

Both CBL (Mean difference −222 ml (−337 to −106, 95%CI), *p* < 0.001, Unpaired T test) and percentage drop in Hb (mean difference 4.3% (−6.3 to −2.3, 95%CI), *p* < 0.001, Unpaired *T* test) were significantly lower in the TXA group (Table 2, Fig. 3). Both CBL and percentage drop in Hb differed between fractures types (*p* < 0.001, ANOVA) and operation type (*p* < 0.001, ANOVA) being maximal in patients treated with DHS or cephalomedullary nail (Table 3, Fig. 4). The percentage drop in Hb was significantly less in the TXA group following IC fracture surgery (mean difference −3.1% (−5.2 to −0.9, 95%CI), *p* = 0.005).

**Fig. 5:**
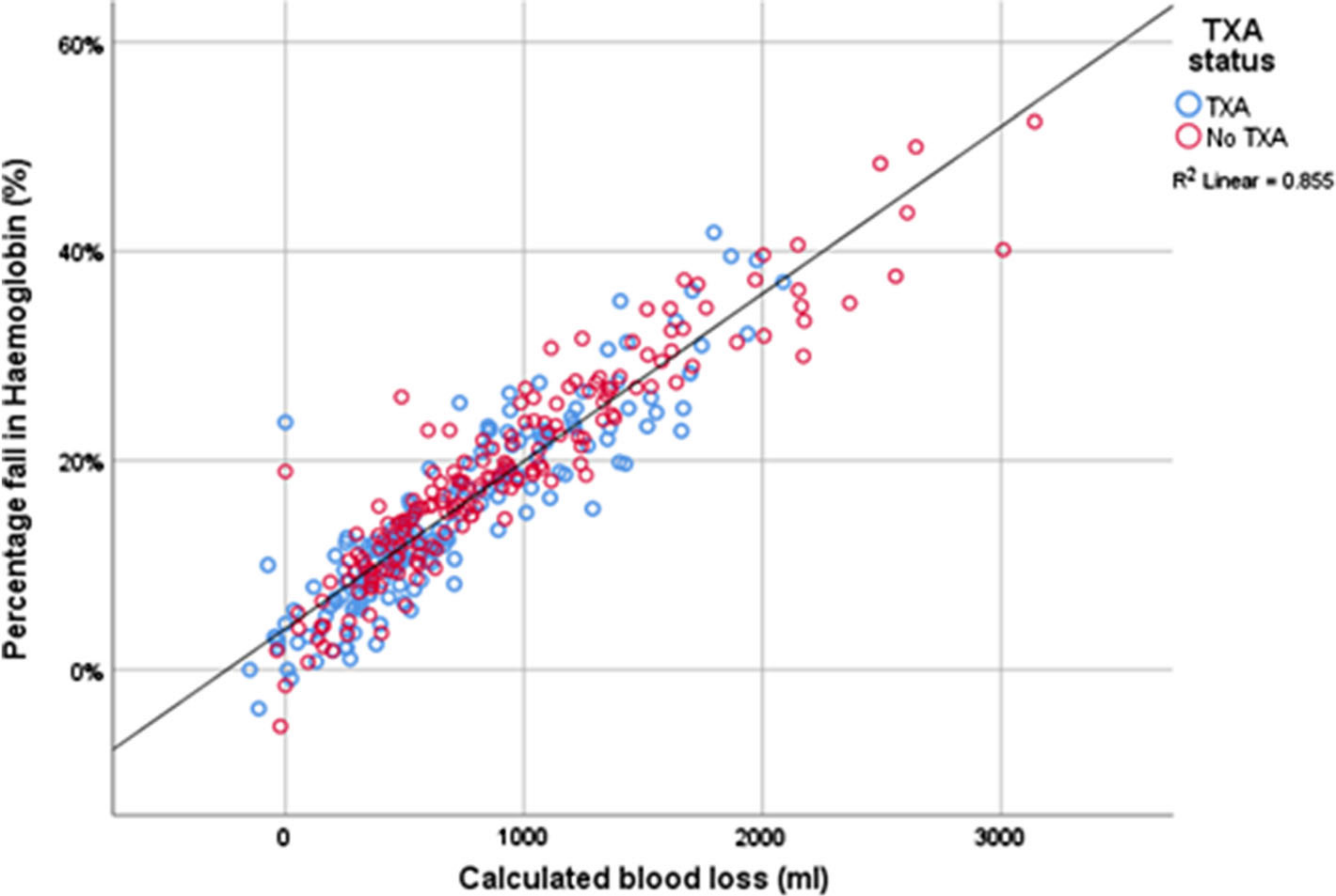
Calculated blood loss (CBL) and percentage drop in haemoglobin (Hb) according to TXA status

**Fig. 6:**
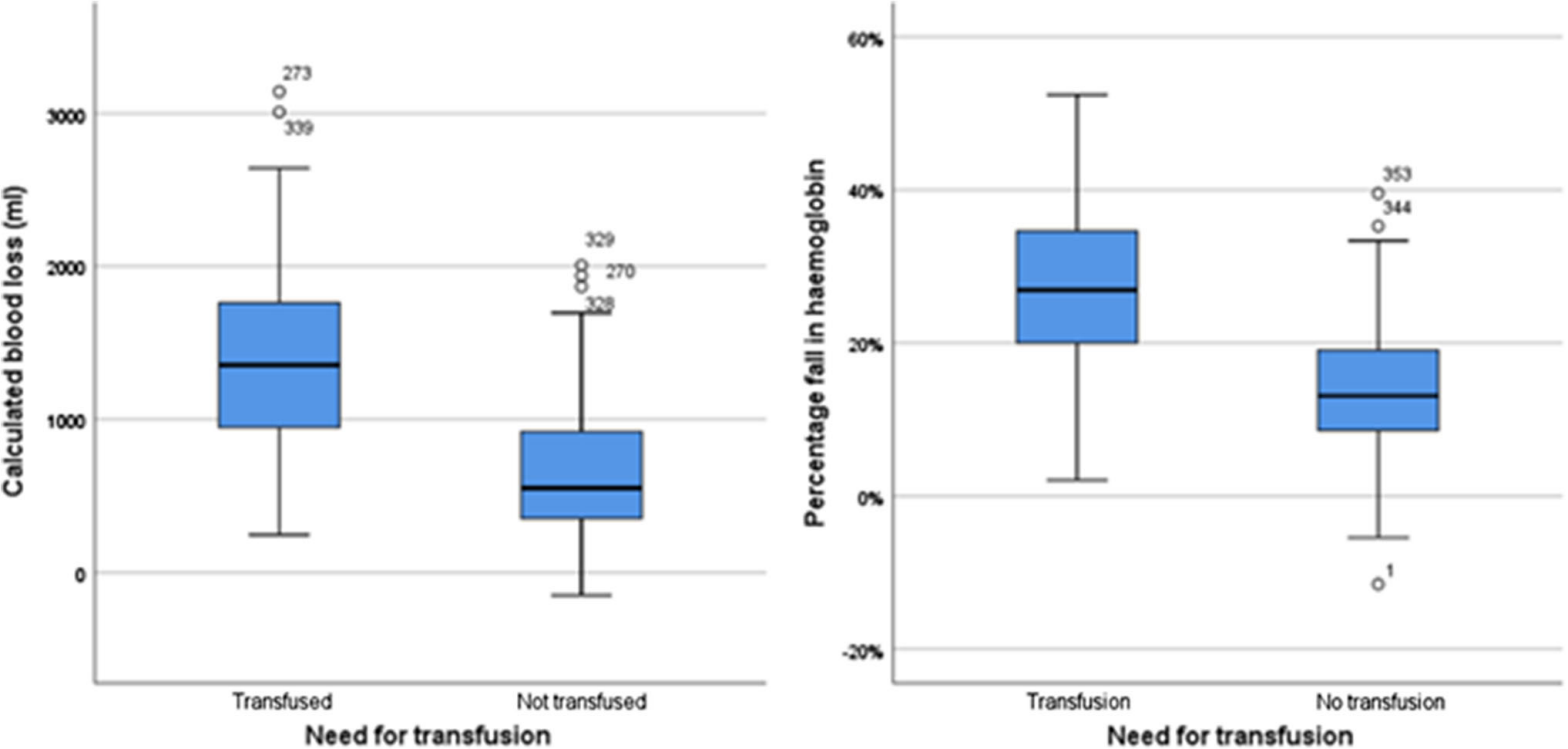
Calculated blood loss (CBL) and percentage drop in haemoglobin (Hb) and the need for blood transfusion

Percentage fall in haemoglobin and CBL correlated significantly suggesting the CBL was reflective of actual blood loss (Pearson’s correlation 0.925, *p* < 0.001, R2 0.855, Fig. 5). Both percentage fall in Hb (Spearman’s rho -0.485, *p* < 0.001) and CBL (Spearman’s rho 0.453, *p* < 0.001) correlated significantly with the need for blood transfusion confirming both to be good measures of actual blood loss (Fig. 6).

### Complications

There was no difference in the rate of complications between the TXA and control groups (Table 4). Specifically, there was no difference in the rate of image proven VTE (2 vs 1 respectively; *p* = 0.620) or other thrombotic events such as myocardial infarction or stroke/transient ischaemic attack (2 vs 0 respectively; *p* = 0.244; Table 4). There was no difference in 30-day mortality rates between case and control groups (14 vs 13 respectively; *p* = 0.620, Chi square).

**Table 4:** Complications by TXA group. Number [%]

Complication	TXA (*n* = 178)	No TXA (*n* = 183)	p value
AKI	10 [6]	18 [10]	0.138^
MI/TIA	2 [1]	0 [0]	0.244^^
VTE	2 [1]	1 [0.5]	0.620^^
Wound leak	15 [8]	24 [13]	0.151^
30-day mortality	14 [8]	13 [7]	0.795^

^ Chi square, ^^ Fisher’s exact

*AKI* acute kidney injury; *MI* myocardial infarction; *TIA* transient ischaemic attack; *VTE* venous thromboembolism

### Cost-effectiveness

Using the rates of transfusion requirement of 15/178 following TXA and 58/183, TXA use resulted in a cost saving of £14,692.50 for the cohort or £82.54 per patient.

## Discussion

To the authors knowledge, this is the first study to investigate the role of TXA in the management of FFF. The data suggest that 1 g of TXA administered intravenously at the start of surgery can significantly reduce the requirement for postoperative blood transfusion. TXA also significantly reduced the calculated blood loss with no apparent detrimental effect on short-term complications or 30-day mortality. The greatest effect of TXA in reducing blood loss and transfusion requirement was in patients treated with DHS and cephalomedullary nails. FFF patients share many similarities in terms of comorbidity and frailty despite having different fracture levels. The apparent beneficial effect of TXA in terms of blood loss, without significant increased thrombotic complications, potentially advocates the incorporation of TXA into FFF treatment guidelines.

Though this is a large prospective case–control study, it is non-randomised, and the findings may have been influenced by selection bias. Some patients who did not receive TXA may have been considered to have contraindications to the use of TXA, such as significant thrombotic disease or risk factors. Both groups included comparable patient numbers who were receiving anticoagulants preoperatively (TXA group 44 vs control group 49) with no significant differences in preoperative comorbidities or ASA grades.

All patients were given the same dose of TXA, and this was not adjusted for weight. Only a single intravenous dose was considered. Dual dosing at the beginning and end of surgery may have a different effect. Though no difference was found in VTE events, the study number was not powered to assess this. All patients were treated according to the department VTE prophylaxis guidelines with Dalteparin (dosing details previously discussed) or restarting of the patient’s normal anticoagulation (e.g. warfarin or apixaban). The follow-up was limited to 30 days and possible effects on mortality beyond that time cannot be commented upon.

Though the current study has not undertaken a complete costs analysis, the reduction in transfusion requirement associated with TXA use led to an estimated cost saving of £82.54 per patient. Considering the high volume and increasing incidence of FFF, this represents a considerable potential annual cost saving: £125,130 for the study unit.

The current study supports previous studies that have demonstrated a beneficial role of TXA in hip fracture surgery without an increase in VTE risk [6, 9, 10, 11, 12, 15, 17, 25]. Despite level 1 evidence for the use of TXA in intracapsular fractures managed with THA or hemiarthroplasty [11, 14] and in extracapsular fractures managed with DHS or CMN [6, 12, 17, 26], a recommendation for its use has not been included in the US or UK national guidelines [18, 21, 25]. RCTs have provided evidence for the use of TXA, but these studies have all included one particular fracture type or one particular type of surgery (e.g. short nails) [6, 12, 17, 26]. These narrow inclusion criteria make them less generalisable to the entire FFF population and incorporation into comprehensive FFF guidelines difficult [18, 21, 25, 27, 28].

Our study does not address the dose of TXA to be used. At standard doses (10–15 mg/kg), TXA inhibits conversion of plasminogen to plasmin, when administered at higher doses (20–25 mg/kg) it will also act directly on plasmin increasing its antifibrinolytic effect [28]. TXA pharmacokinetics include minimal hepatic metabolism and 95% of the dose is excreted renally. The half-life of TXA in normal healthy adult is 2.3 h [29]. TXA dose depends on clinical indication; doses to prevent blood loss (menorrhagia) 1–1.5 g orally a day, major traumatic event IV 1 g bolus followed by Ig infusion over eight hours, post-partum haemorrhage IV 4 g bolus, followed by further doses up to 10 g in 24 h, elective THA 15 mg/kg (approximately 1 g) [29]. The dose reflects the risk benefit ratio of the clinical indication and the potency of the TXA. If a patient has renal failure or an acute renal injury, this will influence the half-life and therefore the effect of the TXA [29].

Currently, the European Society of Anaesthetists does not recommend TXA use for patients requiring hip fracture surgery [27, 28, 30, 31, 32, 33]. The reluctance to use TXA in this frail population is multi-factorial. These patients have multiple comorbidities often with ischaemic heart disease, previous stroke, or hypertension [28, 32]. Fluid depletion on admission with associated acute kidney injuries is common [28]. With this clinical situation, there is a concern that TXA even at a low dose could be clot promoting [26, 25, 27, 28, 29, 30, 31, 32]. There is some evidence that TXA use is an independent risk factor for VTE in trauma patients [33]. Concern persists that frail elderly patients may be in a hypercoagulable state and that a single dose of TXA could cause an increase in clinically significant VTE events [31–33]. This study found no statistical difference in thromboembolic events between the study groups; however, more patients in the TXA group suffered a postoperative thromboembolic event (4/178 vs 1/183). The failure to meet statistical significance may represent a type 2 error. Population- or registry-based studies are required to provide the power to investigate these rare complications. Reassuringly, a recent study of 3097 patients has demonstrated equivalent mortality and fewer thromboembolic event in patients receiving perioperative TXA following a regional change in practice from no TXA to perioperative TXA in patients with hip fracture [34].

## Conclusion

Though the study is not randomised, it has demonstrated that intraoperative intravenous TXA during the surgical management of FFF was associated with significantly less blood loss compared to patients who did not receive TXA. There was no evidence of increased complications, and TXA used in this way appears to be a cost-effective intervention.
